# Heterozygous Recurrent Mutations Inducing Dysfunction of *ROR2* Gene in Patients With Short Stature

**DOI:** 10.3389/fcell.2021.661747

**Published:** 2021-04-14

**Authors:** Baoheng Gui, Chenxi Yu, Xiaoxin Li, Sen Zhao, Hengqiang Zhao, Zihui Yan, Xi Cheng, Jiachen Lin, Haiyang Zheng, Jiashen Shao, Zhengye Zhao, Lina Zhao, Yuchen Niu, Zhi Zhao, Huizi Wang, Bobo Xie, Xianda Wei, Chunrong Gui, Chuan Li, Shaoke Chen, Yi Wang, Yanning Song, Chunxiu Gong, Terry Jianguo Zhang, Xin Fan, Zhihong Wu, Yujun Chen, Nan Wu

**Affiliations:** ^1^Center for Medical Genetics and Genomics, The Second Affiliated Hospital of Guangxi Medical University, Nanning, China; ^2^The Guangxi Health Commission Key Laboratory of Medical Genetics and Genomics, The Second Affiliated Hospital of Guangxi Medical University, Nanning, China; ^3^Department of Pediatric Endocrine and Metabolism, Maternal and Child Health Hospital of Guangxi, Nanning, China; ^4^Department of Orthopedic Surgery, Peking Union Medical College Hospital, Peking Union Medical College and Chinese Academy of Medical Sciences, Beijing, China; ^5^Beijing Key Laboratory for Genetic Research of Skeletal Deformity, Beijing, China; ^6^Medical Research Center, Peking Union Medical College Hospital, Peking Union Medical College and Chinese Academy of Medical Sciences, Beijing, China; ^7^Department of Pediatrics, The Second Affiliated Hospital of Guangxi Medical University, Nanning, China; ^8^Department of Endocrinology, Genetics and Metabolism, Beijing Children’s Hospital, National Center for Children’s Health, Capital Medical University, Beijing, China; ^9^State Key Laboratory of Complex Severe and Rare Diseases, Peking Union Medical College Hospital, Chinese Academy of Medical Sciences and Peking Union Medical College, Beijing, China; ^10^Key Laboratory of Big Data for Spinal Deformities, Chinese Academy of Medical Sciences, Beijing, China

**Keywords:** short stature, *ROR2* gene, recurrent mutations, dysfunction, skeletal development

## Abstract

**Purpose:**

ROR2, a member of the ROR family, is essential for skeletal development as a receptor of Wnt5a. The present study aims to investigate the mutational spectrum of *ROR2* in children with short stature and to identify the underlying molecular mechanisms.

**Methods:**

We retrospectively analyzed clinical phenotype and whole-exome sequencing (WES) data of 426 patients with short stature through mutation screening of *ROR2*. We subsequently examined the changes in protein expression and subcellular location in *ROR2* caused by the mutations. The mRNA expression of downstream signaling molecules of the Wnt5a–ROR2 pathway was also examined.

**Results:**

We identified 12 mutations in *ROR2* in 21 patients, including 10 missense, one nonsense, and one frameshift. Among all missense variants, four recurrent missense variants [c.1675G > A(p.Gly559Ser), c.2212C > T(p.Arg738Cys), c.1930G > A(p.Asp644Asn), c.2117G > A(p.Arg706Gln)] were analyzed by experiments *in vitro*. The c.1675G > A mutation significantly altered the expression and the cellular localization of the ROR2 protein. The c.1675G > A mutation also caused a significantly decreased expression of c-Jun. In contrast, other missense variants did not confer any disruptive effect on the biological functions of ROR2.

**Conclusion:**

We expanded the mutational spectrum of *ROR2* in patients with short stature. Functional experiments potentially revealed a novel molecular mechanism that the c.1675G > A mutation in *ROR2* might affect the expression of downstream Wnt5a–ROR2 pathway gene by disturbing the subcellular localization and expression of the protein.

## Introduction

As a member of ROR family receptor tyrosine kinase, receptor tyrosine kinase like orphan receptor 2 (ROR2) is a 943-amino acid transmembrane protein tyrosine kinase encoded by the *ROR2* gene (van Bokhoven et al., 2000). The extracellular domains of ROR2 mainly include an immunoglobulin-like functional domain, a cysteine enrichment domain (CRD), and a kringle domain, while the intracellular domains include a tyrosine kinase domain (TKD), a proline-rich domain, two serine/threonine-rich domains, and a short C-terminal tail (Endo and Minami, 2018; Kamizaki et al., 2020). ROR2 is widely expressed in a variety of tissues, including the heart, brain, and lung and is also involved in the development of the nervous system and the skeletal system (Huang et al., 2015). Recently, it has been reported that *ROR2* and *ROR1* interact with Wnt9a to regulate the growth of the humerus *in vivo* (Weissenböck et al., 2019).

Pathogenic mutations in *ROR2* are involved in two diseases: autosomal recessive Robinow syndrome (RRS, MIM:268310) and autosomal dominant brachydactyly type B1 (BDB1, MIM:113000) (Afzal et al., 2000; Yang et al., 2020; Zhang et al., 2020). Variants of *ROR2* that cause RRS are generally nonsense, missense, and frameshift and are located in all of the domains (Kirat et al., 2020). BDB1-related variants are often nonsense or frameshift variants that reside in the N-terminal region of the protein (Stricker et al., 2017). Truncating variants associated with BDB1 cause a gain-of-function effect, whereas RRS-related variants result in the loss of function of *ROR2* (Bacino, 1993). Accumulating evidence from *Ror2*-knockout mice and RRS patients suggests a significant role of ROR2 in the early formation of chondrocytes as well as the development and formation of bone (DeChiara et al., 2000; Takeuchi et al., 2000; Patton and Afzal, 2002; Schwabe et al., 2004; Weissenböck et al., 2019).

Recent findings indicate that mutations in a single gene, such as *SHOX*, *NPR2*, *ACAN*, or *FGFR3*, could cause either severe skeletal dysplasia or isolated short stature. Hauer et al. (2018) performed whole-exome sequencing (WES) on 200 patients with idiopathic short stature and found a proportion of patients with pathogenic mutation in genes known to be associated with skeletal dysplasia. We previously revealed distinct genetic architecture and pathophysiological processes in 561 patients with isolated and syndromic short stature using WES and yielded a diagnostic rate of 24.1% (Fan et al., 2021). The milder phenotype (that is, isolated short stature) tends to occur when the mutation only partially disrupts protein function and/or when the mutation occurs in the heterozygous state (Baron et al., 2015).

Although mutations in *ROR2* have been implicated in certain congenital skeletal defects, including BDB1 and RRS, the molecular mechanisms underlying isolated short stature still remain elusive. This study aimed to explore the contributions of heterozygous *ROR2* variants in short stature patients that remained undiagnosed after WES analysis. We subsequently performed *in vitro* functional analyses for variants that were recurrent in our cohort.

## Materials and Methods

### Cohort Recruitment and Whole-Exome Sequencing

From July 2014 to August 2018, we screened 426 WES-undiagnosed patients from three centers in China [Maternal and Child Health Hospital of Guangxi, The Second Affiliated Hospital of Guangxi Medical University, and Beijing Children’s Hospital, as parts of the Deciphering Disorders Involving Scoliosis and COmorbidities (DISCO)] study^1^.

DNA was extracted from peripheral blood collected from all of the probands and available familial members. In total, 374 patients underwent proband-only WES, while 50 underwent trio-based WES, and two underwent quad-based WES (altogether 532 subjects). The sequencing data were analyzed and annotated using an in-house developed analytical pipeline, Peking Union Medical College Hospital Pipeline (PUMP), as previously described (Wang et al., 2018; Chen et al., 2021; Zhao et al., 2021; Supplementary Methods).

### Mutation Analysis

**FIGURE 1:**
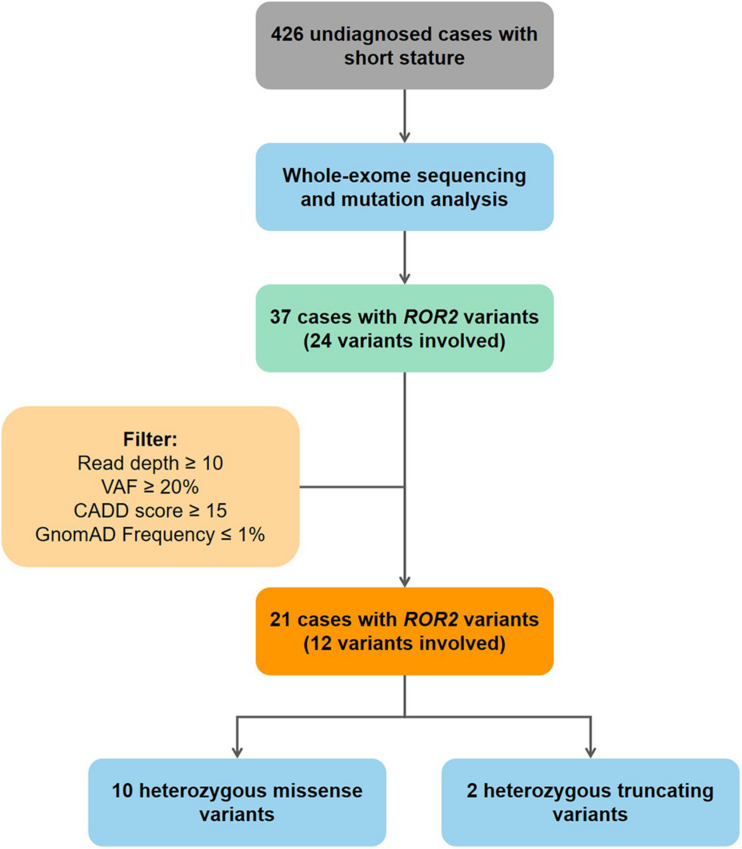
Flowchart of patient enrollment and mutation screening. VAF, variant allele frequency; CADD, combined annotation dependent depletion database.

Mutation screening of *ROR2* was performed in 426 undiagnosed patients. All mutations in coding exons of *ROR2* from our cohort were manually reviewed using Integrative Genomics Viewer (IGV) (Thorvaldsdóttir et al., 2013). Current study use followed criteria to ensure that mutations in *ROR2* were highly credible: (1) read depth of mutation > 10; (2) variant allele frequency ≥ 0.2; (3) Combined Annotation Dependent Depletion (CADD) predicted score > 15; (4) frequency in Gnomad database ≤ 0.01 (Figure 1). Sanger sequencing was performed on available subjects and parental samples to validate the variants by an orthogonal sequencing method and to investigate segregation according to Mendelian expectations for the identified variant allele(s).

### Plasmid Construction

The wild-type (WT) ROR2 plasmid was constructed through Seamless Cloning reaction. The resulting PCR amplimers were digested with *Xho*I and *Bam*HI and ligated into the *Xho*I and *Bam*HI sites of plasmid pEGFP-N1 (Beijing Hitrobio Biotechnology Co., Ltd., Sigma-Aldrich, St. Louis). The resulting plasmid was identified by Sanger sequencing (Forward primer: 5′-CGCTTTGTCCTTCAGCGTTT-3′; Reverse primer: 5′-AATGCCCCTCATTAACCAGC-3′). All *ROR2* mutated plasmids were constructed using the strategy of homologous recombination (Primers used for mutated plasmid construction were listed in Supplementary Materials). The resulting plasmid was transformed into *Escherichia coli* and validated by Sanger sequencing.

### Cell Culture and Transfection

The HeLa cells were cultured in Dulbecco’s modified Eagle media/High Glucose (Hyclone), supplemented with 5% fetal bovine serum (FBS; 10099141C, Gibco), 100 U/ml penicillin-streptomycin (15140122, Gibco) at 37°C with 5% CO_2_. Cells were seeded in six-well plates or confocal dishes and transfected with Lipofectamine 3000 Transfection Reagent (2264840, Invitrogen) according to the manufacturers’ instructions. Six hours after transfection for Western blotting (WB) analysis, the HeLa cells were treated with 100 ng/ml Wnt5a (645-WN-010, R&D Systems) for 48 h.

### Immunofluorescence

The HeLa cells were implanted on confocal dishes and transfected with plasmids using Lipofectamine 3000 Transfection Reagent (2264840, Invitrogen) for 48 h. The confocal dishes were rinsed three times with phosphate-buffered saline (PBS; SH0021, Beijing Haicheng Yuanhong Technology Co., Ltd.). The cells were fixed in 4% fixative solution (P1110, Solarbio, China) and blocked with bovine serum albumin buffer (ZLI-9022, ZSGB-bio, China). The first antibody (rabbit polyclonal calnexin antibody, ab22595, Abcam) was incubated with PBS containing 0.1% Triton X-100 (T8200, Solarbio, China) at room temperature (RT; 25°C) for 1 h. Goat anti-rabbit IgG H&L (Alexa Fluor 647) was adopted as secondary antibody (ab150079, Abcam). The dyed dishes were mounted in fluorescent mounting medium with 4′,6-diamidino-2-phenylindole (DAPI; ZLI-9557, ZSGB-BIO, China) and observed using confocal microscopy. All experiments were replicated three times.

### Western Blotting Analysis

Cells were lysed with radioimmunoprecipitation assay (RIPA) lysis buffer (C1053, Beijing Applygen Technologies Inc.), and protein concentrations were determined using the bicinchoninic acid (BCA) Protein Assay Kit (PC0020, Solarbio, China). Total protein of 20 μg was separated on a 10% NuPAGE^TM^ Bis-Tris Welcome Pack (NP030B, Invitrogen), and the electrophoresed products were transferred to iBlot^TM^ 2 NC Regular Stacks (IB23001, Life Technologies). Membranes were blocked for 30 min at RT using 5% powdered milk, and primary antibodies mouse anti-eGFP mAb (TA-06, ZSGB-BIO, China) and mouse anti-GAPDH mAb (TA-08, ZSGB-BIO, China) were incubated overnight at 4°C. After washing the membranes, the secondary antibody Goat anti-Mouse IgG (H + L) (ZB-2305, ZSGB-BIO, China) was incubated for 2 h at RT. Bands were visualized with Pro-light HRP substrate chemiluminescent system (PA112, Tiangen Biotech Co., Ltd.). Chemiluminescent signals were quantified using ImageJ software.

### Quantitative Real-Time Polymerase Chain Reaction

The levels of c-Jun and Axin2 mRNA in HeLa cells after Wnt5a treatment were analyzed *via* qRT-PCR. Total RNA was extracted using FastPure cell/tissue total RNA isolation kit V2 (RC112-01, Nanjing Vazyme Biotech Co., Ltd.), according to the manufacturer’s instructions. The RNA was subsequently reverse transcribed to yield single-stranded cDNAs using PrimeScript^TM^ RT reagent kit with gDNA eraser (perfect real time, RR047A, TaKaRa) based on the manufacturer’s instructions. The qRT-PCR reaction was performed using TB Green Premix Ex Taq^TM^ II (Tli RNaseH Plus, RR820A, TaKaRa) and the 7500 Fast Dx Real-Time PCR Instrument (Applied Biosystems, United States) based on the manufacturer’s instructions. The primer sequences used for PCR amplification in our study were designed based on the sequences of the cDNA clones as follows: the primers of c-Jun were as previously described (Davoulou et al., 2020) and the primers of Axin2 (137 bp: NM_011359): Forward primer: 5′-CGATGAGTTTGCCTGTGGAG-3′; Reverse primer: 5′-TCAATCGATCCGCTCCACTT-3′.

### Statistical Analysis

Comparison among groups was performed by the analysis of Student’s *T*-test. *P*-value less than 0.05 was statistically significant as calculated by SPSS 21.0.

## Results

### Cohort Demographic Characteristics

We found that 21 patients carried variants in *ROR2* from 426 patients with short stature. Among these patients, there were 12 males (57%) and 9 females (43%). The mean age, bone age, and height SDs of all cases with *ROR2* variants were 6.38 ± 3.99 years, 4.67 ± 2.19 years, and −2.92 ± 1.31, respectively. Ten cases presented other systemic symptoms besides short stature. Six cases presented symptoms of developmental delay, and only one case had a symptom of gonadal dysplasia (Table 1).

**TABLE 1:** Phenotype and genotype of patients carrying mutations in *ROR2.*

ID	Gender	CA, years	BA, years	Height, SDs	Phenotypes	HGVS nomen- clature	Genomic position^#^	Mutational type	Zygosity	Exon	Domain	ExAC frequency*	Gnomad frequency**	CADD score
DISCO-S0061	M	12.00	9.00	−4.00	Intellectual disability HP:0001249; Webbed penis HP:0030264; Abnormality of the testis size HP:0045058	c.1675G > A(p.Gly559Ser)	94487101	Missense	Het	exon9	Protein kinase- Tyrosine	0.0032	0.0037	15.35
DISCO-S0097	M	6.00	NA	NA	Short stature HP:0004322; Delayed speech and language development HP:0000750; Intellectual disability HP:0001249; Motor delay HP:0001270; Wide nasal ridge HP:0012811; Visual impairment HP:0000505									
DISCO-S0024	F	6.50	5.00	−4.00	Global developmental delay HP:0001263; Microcephaly HP:0000252									
DISCO-S0064	F	4.42	2.50	−4.00	NA									
DISCO-S0303	F	2.92	1.50	−3.00	NA									
DISCO-S0098	M	7.42	5.00	−2.50	NA	c.2212C > T(p.Arg738Cys)	94486564	Missense	Het	exon9	Protein kinase- Tyrosine	0.0024	0.0024	16.08
DISCO-S0180	F	9.08	8.00	−2.50	NA									
DISCO-S0189	M	14.00	NA	−3.20	NA									
DISCO-S0126	M	0.67	NA	NA	Short stature HP:0004322; Ectopic kidney HP:0000086									
DISCO-S0874	M	4.58	3.00	−2.13	NA	c.1930G > A(p.Asp644Asn)	94486846	Missense	Het	exon9	Protein kinase- Tyrosine	0.0017	0.0019	22.8
DISCO-S0891	M	3.92	3.00	−2.74	NA									
DISCO-S0158	M	5.83	3.50	−3.80	NA	c.2117G > A(p.Arg706Gln)	94486659	Missense	Het	exon9	Protein kinase- Tyrosine	0.0041	0.0045	18.45
DISCO-S0060	M	9.50	NA	−2.00	Abnormality of the pituitary gland HP:0012503									
DISCO-S0098	F	0.17	NA	−3.00	Motor delay HP:0001270; Muscular hypotonia HP:0001252; Blepharophimosis HP:0000581; Feeding difficulties HP:0011968	c.935G > A(p.Arg312His)	94495406	Missense	Het	exon6	Kringle-like fold	0.0064	0.0035	15.75
DISCO-S0134	F	0.29	NA	−2.00	Motor delay HP:0001270; Increased lactate dehydrogenase activity HP:0025435	c.553T > C(p.Phe185Leu)	94499742	Missense	Het	exon5	Frizzled	0	0	15.21
DISCO-S0023	F	7.50	5.00	−3.80	Global developmental delay HP:0001263; Microcephaly HP:0000252	c.302C > T(p.Pro101Leu)	94519715	Missense	Het	exon3	Immuno- globulin	0.0032	0.0037	15.35
DISCO-S0204	M	6.75	5.50	−4.00	NA	c.769G > A(p.Glu257Lys)	94495572	Missense	Het	exon6	Frizzled	0.00024	0.0002	36
DISCO-S0292	F	3.50	NA	−5.00	NA	c.2236C > T(p.Leu746Phe)	94486540	Missense	Het	exon9	Protein kinase- Tyrosine	0	0	15.29
DISCO-S0120	F	7.00	NA	1.10	Motor delay HP:0001270; Intellectual disability HP:0001249	c.613C > T(p.Arg205Ter)	94499682	Stop gain	Het	exon5	Frizzled	0	0	40
DISCO-S0042	M	14.30	NA	−2.00	Abnormality of the hypothalamus–pituitary axis HP:0000864	c.2014G > A(p.Asp672Asn)	94486762	Missense	Het	exon9	Protein kinase- Tyrosine	0	0	17.72
DISCO-S0186	M	8.70	5.00	−2.80	NA	c.2625dupC (p.Thr876fsTer20)	94486150	Frameshift	Het	exon9	Ser/Thr-rich region	0	0	NA

^#^Genomic position was based on GRCh37 Chromesome 9. *Frequency was derived from the East-Asian frequency of Exome Aggregation Consortium (ExAC) database. **Frequency was derived from the East-Asian frequency of Genome Aggregation Database (Gnomad).

*HGVS, Human Genome Variation Society; CADD, Combined Annotation Dependent Depletion; Het, heterozygous; NA, not available; CA, chronological age; BA, bone age; SD, standard deviation; F, female; M, male; HP, Human Phenotype Ontology (HPO) term identifier.*

### Mutational Screening of ROR2 in the Cohort

**FIGURE 2:**
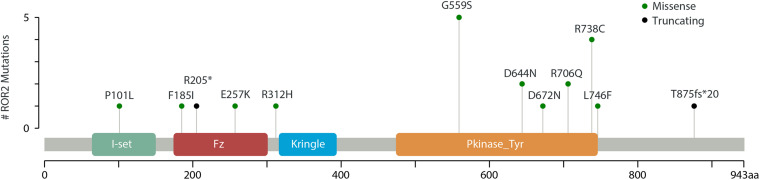
Schematic representation of *ROR2* with its domain structure. Mutations identified in this study (green and black) are marked.

Through mutation screening analysis, we identified 12 mutations in *ROR2*, including two truncating mutations and 10 missense mutations (Table 1). The heterozygous stop-gain mutation [c.613C > T (p.Arg205Ter)] was found in the patient DISCO-S0120 who manifested dwarfism with mild motor retardation and mental retardation, which were partly consistent with the phenotype of RRS. The other patient DISCO-S0186 carried a heterozygous *ROR2* frameshift mutation [c.2625dupC (p.Thr876fsTer20)], which was absent from pubic databases and 942 in-house control from the DISCO study. However, no variant in *ROR2* was identified *in trans* in either patient. Furthermore, four recurrent missense mutations in *ROR2* were identified, including c.1675G > A (p.Gly559Ser) in five patients, c.2212C > T (p.Arg738Cys) in four patients, c.1930G > A (p.Asp644Asn) in two patients, and c.2117G > A (p.Arg706Gln) in two patients (Table 1). All recurrent missense mutations were located at the TKD of the intracellular region of the ROR2 protein (Figure 2).

### Expression of the ROR2 Protein and Wnt5a–ROR2 Pathway-Related Protein

**FIGURE 3:**
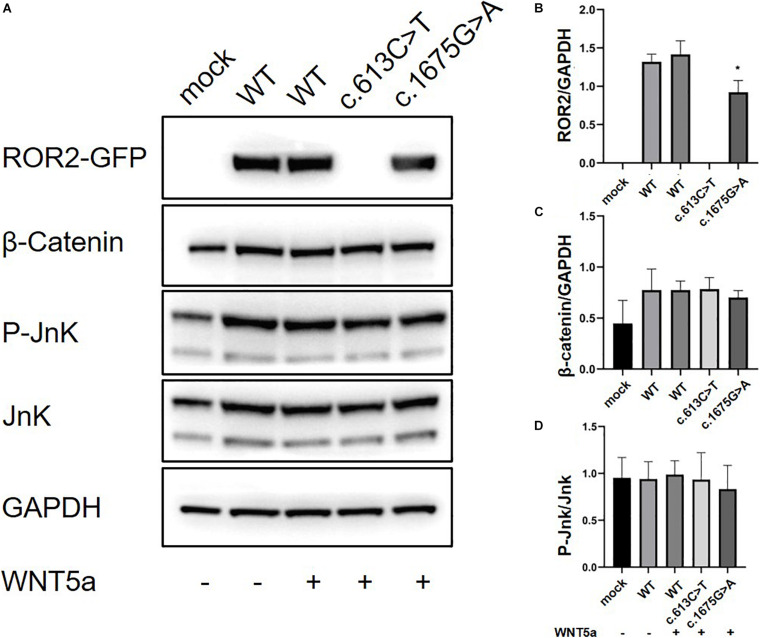
Modulation of non-canonical Wnt signaling in HeLa cells with the supplementation of Wnt5a in general culture conditions. The ROR2-GFP, β-Catenin, P-JnK, JnK levels in HeLa cells with 100 ng/ml concentration of Wnt5a **(A)** Western blot for Ror2 [wild type (WT) and mutants] in HeLa cells. **(B–D)** Quantification of protein expression of different *ROR2* constructs by ImageJ (*n* = 3), *P < 0.05 vs. WT + Wnt5a 100 ng/ml.

**FIGURE 4:**
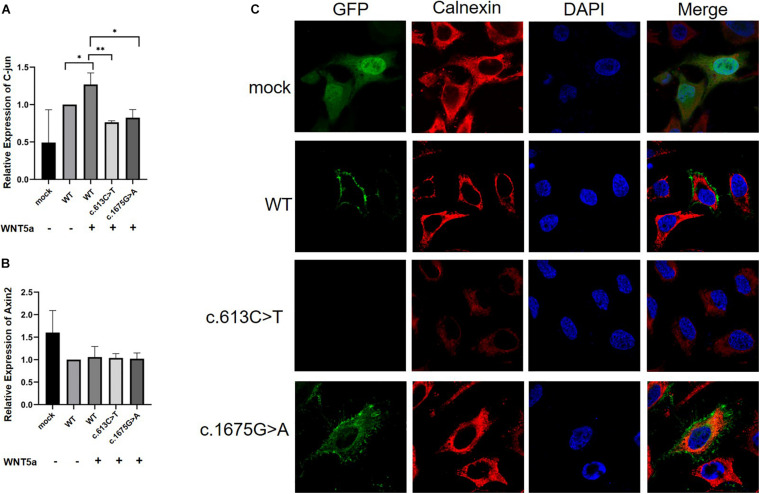
Real-time PCR analysis of mRNA expression of Wnt ligands (C-Jun and Axin2) in HeLa cells (*n* = 3) and subcellular location of the ROR2 protein. All *P*-values were calculated by Student’s *t*-test. ^∗^*P* < 0.05 and ^∗^*P* < 0.01 were considered statistically significant. **(A)** qPCR analysis of C-jun mRNA expression under Wnt5a treatment. **(B)** qPCR analysis of Axin2 mRNA expression under Wnt5a treatment. **(C)** Subcellular location distribution of ROR2 WT protein, and distribution of mutated ROR2 protein.

We further conducted *in vitro* protein expression experiments to examine functional defects caused by recurrent missense mutations in *ROR2*. WB showed that c.1675G > A mutation could lead to a significant decrease in the expression of ROR2 protein with Wnt5a treatment (*P* < 0.05; Figures 3A,B). In contrast, the remaining three missense variants did not impact the expression of ROR2 (data not shown). The influence of mutated and WT ROR2 protein on β-catenin and P-Jnk/JNK pathways showed no significant difference with or without Wnt5a treatment (Figures 3A,C,D). To further explore the effect on the Wnt5a pathway conferred by the c.1675G > A variant, we tested the expression of c-Jun, a molecule downstream of the Wnt5a pathway in precursors of osteoclast (Maeda et al., 2012), and Axin2, a regulator of the Wnt pathway, with Wnt5a stimulation (Lammi et al., 2004). As a result, mRNA expression of c-Jun in the cells transfected with mutated *ROR2* c.1675G > A was significantly lower than that transfected with WT *ROR2* (*P* < 0.05; Figure 4A). In contrast, there was no significant difference in the expression of Axin2 mRNA after treatment with Wnt5a (Figure 4B).

### Immunofluorescence Colocalization of ROR2

We further performed immunofluorescence experiments to explore the effects of these missense mutations on the subcellular localization of the ROR2 protein. The results showed that the mutated *ROR2* c.1675G > A was enriched in the endoplasmic reticulum, and the mutated *ROR2* c.613C > T was not detected (Figure 4C). The subcellular localization of other missense mutations in *ROR2* was similar to that of WT (data not shown). These *in vitro* results suggested that the c.1675G > A mutation might affect the expression of downstream genes in the Wnt5a–ROR2 pathway and alter the subcellular localization of the ROR2 protein.

## Discussion

With the application of the next-generation sequencing (NGS) in exploring the genetic etiology of disease, complex genetic patterns may explain the genetic causes of many common diseases and relatively rare diseases (Posey et al., 2017). Through mutation screening of *ROR2*, we found two patients with heterozygous truncating mutations in *ROR2* mainly presenting short stature. Genotype–phenotype evaluation confirmed that these two patients were lacking other characteristic features of the RRS, indicating that heterozygous loss-of-function mutation in *ROR2* might be associated with isolated short stature.

In addition, we identified six missense mutations in *ROR2* including four recurrent missense mutations located within the TKD (Figure 2 and Table 1). Results from functional experiments *in vitro* demonstrated that the missense mutation in *ROR2* [c.1675G > A (p.Gly559Ser)] could perturb the subcellular localization of the ROR2 protein and lead to a decreased expression of downstream molecule of the Wnt5a pathway. Based on these results, we inferred that this heterozygous missense mutation in *ROR2* might have a hypomorphic effect, which meant a variant caused partial loss of the gene function (Hrabé de Angelis and Balling, 1998).

In vertebrates, ROR2 and ROR1 bind to non-classical Wnt5a protein through their CRD and act as receptors or co-receptors for Wnt5a to activate non-classical Wnt pathways (β-catenin-independent pathways), including planar cellular polarization pathways and Wnt–Ca^2+^ pathways (Oishi et al., 2003; Yamamoto et al., 2007; Gao et al., 2011; Kamizaki et al., 2020). Recently, studies showed that the Wnt5a–ROR2 pathway was not only involved in the development of cartilage but also associated with the function of osteoblasts and osteoclasts (Chen et al., 2019; Uehara et al., 2019). Weissenböck et al. (2019) found that knockout of *Ror2* also influenced the formation of the trunk bone *in vivo*. Maeda et al. (2012) found that the Wnt5a–ROR2 pathway could further regulate the differentiation of osteoblast progenitor cells by affecting the dimer of Jun and Sp-1 binding to the transcriptional initiation region of *Tnfrsf11a*, which is consistent with our findings. According to our *in vitro* assays, c.1675G > A mutation in *ROR2* leads to a decrease in the expression of c-Jun under Wnt5a treatment, which indicated that this missense mutation in *ROR2* might disrupt the normal function of the Wnt5a–ROR2 pathway.

The TKD is the key domain in the cytoplasmic region of the ROR2 protein. The biological function of the TKD was controversial in previous studies (Alfaro et al., 2015; Debebe and Rathmell, 2015). In terms of evolutionary biology, mammalian ROR2 exhibits alterations within the highly conserved amino acids in the kinase domains that possibly indicates that the kinase activity may have been evolutionarily degenerated (Forrester, 2002). Also, several studies suggested that both ROR1 and ROR2 might be pseudokinases (Gentile et al., 2011; Debebe and Rathmell, 2015). However, recent studies by Nevenzal et al. (2019) and Sheetz et al. (2020) had found the autophosphorylation activity and autoinhibitory interactions of the TKD in the ROR2 protein from the perspective of high-throughput phosphorylation detection and protein structure. Consistent with results from experiments *in vitro*, five of our patients carrying the c.1675G > A heterozygous mutation mainly displayed short stature. As shown by both *in vitro* assays and data from patient cohorts, we confirmed that missense mutation c.1675G > A in the TKD region might be associated with linear growth attenuation among children. However, the function of the TKD needs to be confirmed by further molecular biological experiments. Also, our findings from cohort and *in vitro* results both highlighted a possiblely novel mechanism through which hypomorphic mutation in *ROR2* leads to short stature.

## Conclusion

We expanded the mutational spectrum of *ROR2* in patients with short stature. The c.1675G > A in *ROR2* was recurrently seen in five patients and was revealed to confer a hypomorphic effect on the function and expression of the protein and the normal activity of the Wnt5a pathway.

## Data Availability Statement

The data presented in the study are deposited in the ClinVar repository, and accession number are SCV001499871– SCV001499881.

## Ethics Statement

Approval for the study was obtained from the Ethics Committee at the Maternal and Child Health Hospital of Guangxi Zhuang Autonomous Region (G-1-1), The Second Affiliated Hospital of Guangxi Medical University [2020-KY(0112)] and Beijing Children’s Hospital (Y-028-A-01). Written informed consent to participate in this study was provided by the participants’ legal guardian/next of kin.

## Author Contributions

CY, SZ, BG, XL, and NW contributed to the conceptualization. CY, BG, ZY, SZ, XC, XL, and NW contributed to data curation. CY, BG, XL, SZ, HeZ, and JL contributed to the formal analysis. SC, TZ, ZW, CGo, BG, and NW contributed to funding acquisition. XL, JL, HaZ, JS, ZheZhao, ZhiZhao, HW, BX, XW, CGu, and CL contributed to the investigation. SC, CL, CGo, XF, YC, and NW contributed resources. ZY, HeZ, and YN contributed the software. CGo, XF, YC, and NW contributed to supervision, writing, review, and editing. HeZ and CY contributed to visualization. CY, BG, XL, and SZ contributed to writing the original draft. All authors provided crucial input on several iterations of the manuscript, and approved the final version.

## Conflict of Interest

The authors declare that the research was conducted in the absence of any commercial or financial relationships that could be construed as a potential conflict of interest.

## Footnotes

1 http://www.discostudy.org/
