# Supplementary material for: Heterozygous Recurrent Mutations Inducing Dysfunction of ROR2 Gene in Patients With Short Stature
Source: Front Cell Dev Biol. 2021 Apr 14;9:661747. doi: 10.3389/fcell.2021.661747 (PMC8080376; doi:10.3389/fcell.2021.661747)
Supplement: Supplementary file 1 [file Data_Sheet_1.docx]

**Supplementary Material**

This supplemental file was intended for publication as a data supplement. The contents include the following:

**Supplementary Methods**

**Supplementary Reference**

**Supplementary Methods**

**Primers used for *ROR2* plasmid construction**

The sequences of primers listed below is used for construction of the mutated ROR2 plasmid:

ROR2-G1675A-F: TACTGTTCGCACAGCGACCTCCACGAATTCCTG

ROR2-G1675A-R: TCGCTGTGCGAACAGTAGCTGAAGATCATGCT

ROR2-C613T-F: AAACTGAATCACAGCGGCCTTCACCATGATCG

ROR2-C613T-R: CCGCTGTGATTCAGTTTTCAATCTCCCCCTGCAT

ROR2-C2212T-F: CCCTGCTTCAAGGACATCCACAGCCGGCTCCG

ROR2-C2212T-R: ATGTCCTTGAAGCAGGGCCGCCGGCTGGGGAA

ROR2-G1930A-F: ATGCCGCCAATTACTACAAGCTGCTGGGGAACT

ROR2-G1930A-R: GTAGTAATTGGCGGCATACACCTCTCGGAAGA

ROR2-G2117A-F: AGATGATCCAGAACCGGCAGGTGCTGCCTTGC

ROR2-G2117A-R: CCGGTTCTGGATCATCTCCACCACATCCTGGT

**DNA extraction and exome sequencing**

For all centers, genomic DNA for 426 undiagnosed patients (including 52 cases along with their healthy family members and 374 singletons) was extracted from peripheral blood lymphocytes. Whole-exome sequencing (WES) was performed on peripheral blood DNA for all participants. DNA samples were prepared in Illumina libraries and then underwent whole-exome capture with the SureSelect Human All Exon V5 (Agilent, USA, n= 234), SureSelect Human All Exon V6 + UTR r2 core design (Agilent, USA, n= 204), and Trueseq DNA Exome (Illumina, USA, n = 95), followed by sequencing on the Illumina HiSeq 4000 platform in 150-bp paired-end reads mode (Illumina, San Diego, CA, USA). The average sequencing depth of our cohort was 80X.

**Annotation pipeline**

The sequencing data were analyzed and annotated using an in-house developed analytical pipeline, Peking Union Medical College hospital Pipeline (PUMP) (1, 2). Paired sequences obtained from each sample were aligned to the GRCh37/hg19 human reference sequence using Burrows-Wheeler Aligner (BWA) with the MEM algorithm. BAM files were generated by Picard. Sequence reads were recalibrated by Realigner Target Creator in Genome Analysis Toolkit (GATK) and sequence variants were called by GATK Haplotype Caller. Annotation of de novo, compound heterozygous, and recessive inherited variants were calculated with Gemini (version 0.19.1) for in silico subtraction of parental variants from the proband’s variants, with accounting for read number information extracted from BAM files. Computational prediction tools (GERP++ (3), Combined Annotation Dependent Depletion (CADD)(4), SIFT(5), and Polyphen-2(6)) were used to predict the conservation and pathogenicity of candidate variants. Population frequency of each variant were obtained from publicly available databases such as the 1000 Genomes Project (<http://www.internationalgenome.org/>), the Exome variant server, NHLBI GO Exome Sequencing Project (ESP) (<http://evs.gs.washington.edu/EVS/>), the Exome Aggregation Consortium (ExAC) (<http://exac.broadinstitute.org/)>, and genome Aggregation Database (gnomAD , <http://gnomad.broadinstitute.org/>).

**Interpretation of genetic variants**

All variants were first filtered against a general population frequency of 0.01 based on 1000 Genomes, ExAC, and gnomAD databases. Intronic/UTR variants outside canonical splicing sites and synonymous variants without functional reports.

Evaluation of the pathogenicity of the variants was based on the American College of Medical Genetics and Genomics (ACMG) guidelines(7). After selection of pathogenic or likely pathogenic alleles, anticipated mode of inheritance associated with the identified genes was then considered. For dominant or X-linked dominant genes, a heterozygous (or hemizygous) variant is sufficient to be potentially disease-causing. For genes usually associated with an autosomal recessive disease trait inheritance, biallelic variants revealed through trio exome sequencing were required to suspect a gene to be disease-causing.

If the observed variant(s) is/are pathogenic and consistent with the expected mode of inheritance, gene-related phenotypes were compared to the patient phenotype. A positive molecular finding is defined when the phenotypic spectrum of the gene could explain the whole clinical presentation of the patient.

**Supplementary Reference**

1. Zhao S, Zhang Y, Chen W, Li W, Wang S, Wang L, et al. Diagnostic yield and clinical impact of exome sequencing in early-onset scoliosis (EOS). *J Med Genet* (2021) 58(1):41-7. Epub 2020/05/10. doi: 10.1136/jmedgenet-2019-106823. PubMed PMID: 32381727.

2. Wang K, Zhao S, Liu B, Zhang Q, Li Y, Liu J, et al. Perturbations of BMP/TGF-beta and VEGF/VEGFR signalling pathways in non-syndromic sporadic brain arteriovenous malformations (BAVM). *J Med Genet* (2018) 55(10):675-84. Epub 2018/08/19. doi: 10.1136/jmedgenet-2017-105224. PubMed PMID: 30120215; PubMed Central PMCID: PMCPMC6161649.

3. Davydov EV, Goode DL, Sirota M, Cooper GM, Sidow A, Batzoglou S. Identifying a high fraction of the human genome to be under selective constraint using GERP++. *PLoS computational biology* (2010) 6(12):e1001025. Epub 2010/12/15. doi: 10.1371/journal.pcbi.1001025. PubMed PMID: 21152010; PubMed Central PMCID: PMCPMC2996323.

4. Kircher M, Witten DM, Jain P, O'Roak BJ, Cooper GM, Shendure J. A general framework for estimating the relative pathogenicity of human genetic variants. *Nat Genet* (2014) 46(3):310-5. doi: 10.1038/ng.2892. PubMed PMID: 24487276; PubMed Central PMCID: PMCPMC3992975.

5. Vaser R, Adusumalli S, Leng SN, Sikic M, Ng PC. SIFT missense predictions for genomes. *Nature protocols* (2016) 11(1):1-9. Epub 2015/12/04. doi: 10.1038/nprot.2015.123. PubMed PMID: 26633127.

6. Adzhubei IA, Schmidt S, Peshkin L, Ramensky VE, Gerasimova A, Bork P, et al. A method and server for predicting damaging missense mutations. *Nature methods* (2010) 7(4):248-9. Epub 2010/04/01. doi: 10.1038/nmeth0410-248. PubMed PMID: 20354512; PubMed Central PMCID: PMCPMC2855889.

7. Richards S, Aziz N, Bale S, Bick D, Das S, Gastier-Foster J, et al. Standards and guidelines for the interpretation of sequence variants: a joint consensus recommendation of the American College of Medical Genetics and Genomics and the Association for Molecular Pathology. *Genet Med* (2015) 17(5):405-24. Epub 2015/03/06. doi: 10.1038/gim.2015.30. PubMed PMID: 25741868; PubMed Central PMCID: PMCPMC4544753.
